# Identification of epithelial-mesenchymal transition-related biomarkers in lung adenocarcinoma using bioinformatics and lab experiments

**DOI:** 10.18632/aging.205159

**Published:** 2023-10-31

**Authors:** Yuanjun Cheng, Yumei Shen, Qianru Fang, Shanzhou Duan, Yifei Wang, Xiaoxiao Dai, Yongbing Chen

**Affiliations:** 1 Department of Thoracic Surgery, The Second Affiliated Hospital of Soochow University, Suzhou, China; 2 Department of Cardiothoracic Surgery, People’s Hospital of Chizhou, Chizhou, China; 3 Department of Operation, The Second Affiliated Hospital of Soochow University, Suzhou, China; 4 Department of Obstetrics, People’s Hospital of Chizhou, Chizhou, China; 5 Department of Pathology, The Second Affiliated Hospital of Soochow University, Suzhou, China

**Keywords:** epithelial-mesenchymal transition, biomarkers, PLEK2, prognosis, autophagy

## Abstract

Background: Lung adenocarcinoma accounts for approximately 40% of lung cancer cases and poses a serious threat to human health. Therefore, there is an urgent need to identify central biomarkers in lung adenocarcinoma.

Methods: We first identified the EMT-associated genes in LUAD based on the TCGA cohort. Then we screened these 90 EMT-associated genes using univariate Cox regression analysis and LASSO regression analysis to develop a prognostic gene signature in the training set. The predictive performance of the gene signature was assessed in the validation set and multiple external test sets using the ROC cure, C index and log-rank tests. RT-PCR, western blot, wound healing assays, and siRNA methods were further used to investigate the role of PLEK2 in tumor behaviors.

Results: Eight genes (CCNB1, PLEK2, DERL3, C1QTNF6, DLGAP5, HMMR, GJB3, and SPOCK1) were eventually selected to develop an eight-gene signature. The 5-year AUC of the gene signature has a robust predictive ability both for predicting overall survival (0.774, 0.756, and 0.669 in the external test sets, respectively), and for progression free survival (0.774, 0.746, and 0.755 in the external test sets, respectively). C-index of the gene signature was 0.961 ± 0.005, 0.916 ± 0.011, and 0.868 ± 0.234 in the external test sets, respectively. Four genes (C1QTNF6, DLGAP5, HMMR, and PLEK2) were identified as key genes in LUAD progression, which were upregulated in the cancerous tissue compared with in the normal tissue (P < 0.001), and correlated with an unwanted prognosis in lung cancer (P < 0.05). PLEK2 was used as an example to explore its effect on LUAD progression *in vitro* using RT-PCR, western blot, CCK8, si-RNA and wound healing assay. Silencing of PLEK2 was shown to reduce proliferative and migrated ability of lung cancer cells via prohibition of autophagy.

Conclusions: This study developed a novel EMT-related gene signature benefiting precision medicine, and identified four pivotal genes which can serve as therapeutic targets in LUAD. Four key genes can serve as molecular targets for patients with LUAD; silencing of PLEK2 was shown to reduce proliferative and migrated ability of lung cancer cells via prohibition of autophagy.

## INTRODUCTION

Lung cancer represents the first cause of cancer-related death worldwide [1], while lung adenocarcinoma is the most common pathological type of lung cancer, accounting for approximately 50% of non-small cell lung cancers [2, 3], with an average 5-year survival rate of 4% for late-stage lung cancer. It is partly due to poor diagnostic techniques and treatment approaches [4, 5]. In the past, the main treatment for lung cancer was surgery and radiotherapy, but for patients with metastatic lung cancer, radiotherapy alone often has limited therapeutic effects [6, 7]. At present, various biomarkers have been widely discovered and applied in the treatment of various types of tumors [8], demonstrating their roles in the field of cancer.

Epithelial-mesenchymal transition (EMT) is a biological process where epithelial cells are transformed into cells with a mesenchymal phenotype through a specific procedure [9]. It plays an important role in embryonic development, chronic inflammation, tissue reconstruction, cancer metastasis and several fibrotic diseases, and is mainly characterized by a reduction in the expression of cell adhesion molecules (e.g., E-calmodulin), the transformation of the cytokeratin cytoskeleton into a wave protein (Vimentin)-dominated cytoskeleton and morphological features of mesenchymal cells [10]. In the process of EMT, epithelial cells lose their epithelial phenotype such as cell polarity and connection to the basement membrane, and acquire a mesenchymal phenotype such as higher migration and invasion, resistance to apoptosis and ability to degrade the extracellular matrix [11]. It is well known that the EMT process involves multiple transcription factors associated with LUAD progression, and therefore, novel prognostic profiles using EMT-related genes could provide better predictive value and risk stratification for cancer therapy [12].

In the current study, with the help of bioinformatics tools, we identified an EMT-associated prognostic signature and constructed a new nomogram based on data from the GEO and TCGA databases. These findings will provide clinicians and researchers with useful prognostic factors for patients with LUAD.

## RESULTS

### Construction of the EMT-associated gene signature

**Figure 1:**
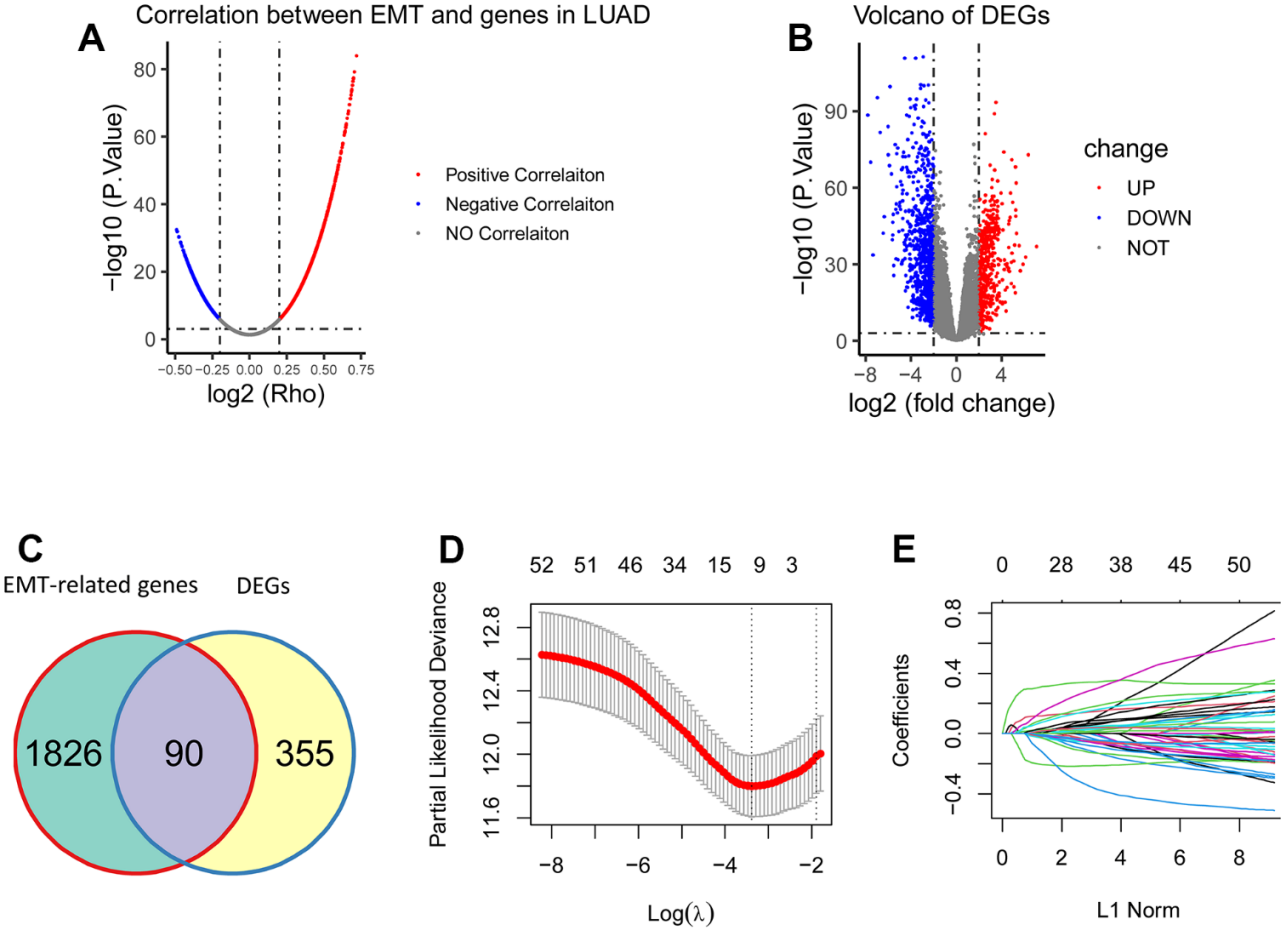
**Construction of the EMT-associated gene signature.** (**A**) 1916 genes were significantly correlated with EMT (*P* < 0.001, *R* > 0.2). (**B**) 1250 DEGs were obtained between tumorous tissues and the matched normal tissues, including 445 upregulated DEGs and 805 downregulated DEGs (|logFC| > 2, FDR < 0.001). (**C**) There were 90 overlapping genes between 1916 EMT-associated genes and 445 upregulated DEGs. (**D**, **E**) 52 genes were screened using LASSO regression analysis, and eight genes (*CCNB1*, *PLEK2*, *DERL3*, *C1QTNF6*, *DLGAP5*, *HMMR*, *GJB3*, and *SPOCK1*) were eventually obtained for construction of the EMT-associated gene signature.

Analysis of the ssGSEA algorithm showed that a total of 1916 EMT-related genes were identified from the TCGA cohort of LUAD patients (P < 0.001, R > 0.2; Figure 1A). The underlying RNA sequencing data (57 paired cancer and normal tissues) was then performed for a total of 1250 DEGs, of which 445 genes were upregulated,805 genes were downregulated (|logFC| > 2, FDR < 0.001; Figure 1B). Among the 1916 EMT-associated genes and 445 up-regulated DEGs, 90 genes were overlapping, and they were eligible EMT-associated genes for establishing gene signatures, and the results showed that a total of 52 genes were eligible because their *P*-values were less than 0.05 (Figure 1C). Then LASSO regression analysis was used to screen these genes, and the results showed that 8 genes (CCNB1, PLEK2, DERL3, C1QTNF6, DLGAP5, HMMR, GJB3 and SPOCK1) could be used to establish 8 gene signatures (Figure 1D, 1E).

### Validation of the EMT-associated gene signature

**Figure 2:**
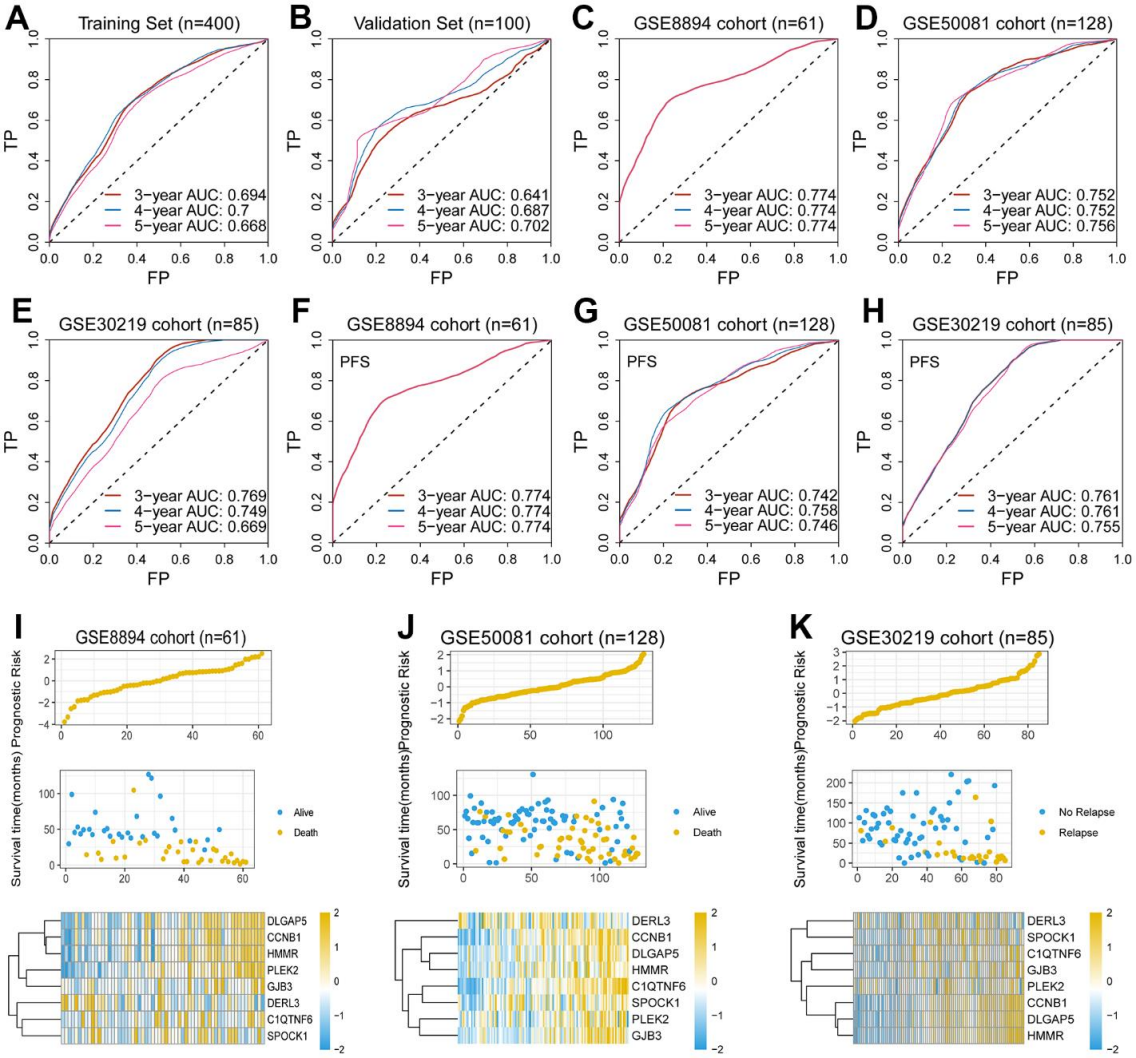
**Validation of the EMT-associated gene signature.** (**A**–**E**) The 5-year AUC of the gene signature for predicting overall survival (OS) was 0.668, 0.702, 0.774, 0.756, and 0.669 in the training set, the validation set and the external test sets, respectively. (**F**–**H**) The 5-year AUC of the gene signature for predicting progression free survival (PFS) was 0.774, 0.746, and 0.755 in three external test sets, respectively. (**I**–**K**) The high-risk scores were associated with poor survival outcomes and high expression levels of the eight genes in the GSE8894 cohort, the GSE50081 cohort, and the GSE30219 cohort.

It was assessed in validation set (n = 100), training set (n = 400) and three independent sets (GSE8894, n = 61; GSE50081, n = 128; GSE30219, n = 85). The 5-year AUC in the above sets was 0.702, 0.668, 0.774, 0.756, and 0.669, respectively (Figure 2A–2E), indicating that this gene signature possesses robust predictive ability for OS.

### Clinical significance of the eight-gene signature

**Figure 3:**
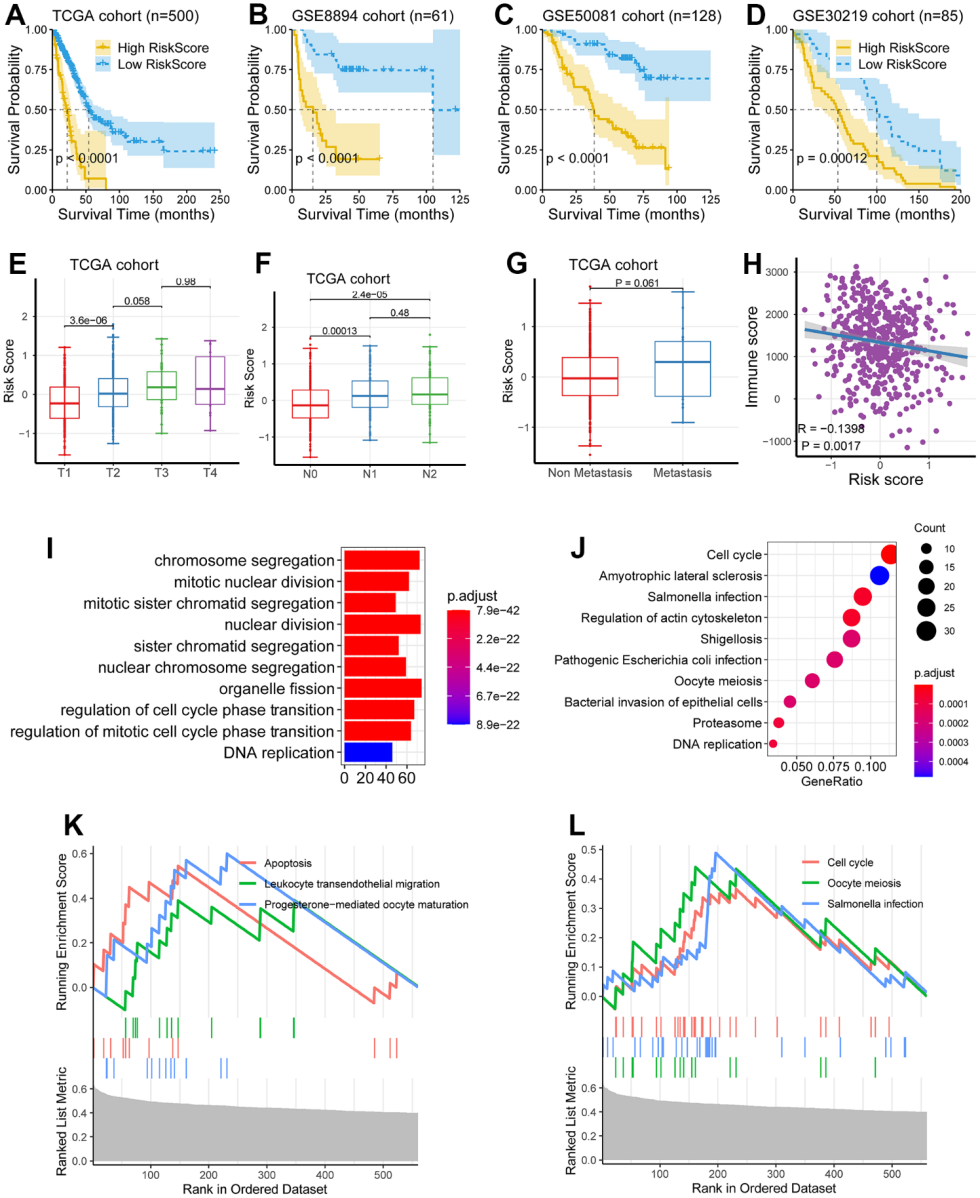
**Clinical significance of the eight-gene signature.** (**A**–**D**) The low-risk group had a better survival time as compared with the high-risk group in the TCGA cohort, the GSE8894 cohort, the GSE50081 cohort, and the GSE30219 cohort. (**E**–**G**) The levels of risk score were significantly augmented in patients with higher TNM staging. (**H**) Risk score was negatively correlated with immune score (*P* = 0.0017, *R* = -0.1398). (**I**–**L**) Multiple biological processes related to tumor progression were significantly enriched, including DNA replication, cell cycle, chromosome segregation, and nuclear division.

To assess the relationship between patient risk scores, a multivariate Cox model based on eight genetic traits was used to calculate the risk-related scores for each patient. The proposed eight genetic traits had a robust predictive power for predicting progression-free survival (PFS), further demonstrating their robust predictive power for survival. In addition, the C-index correlation data of 0.646±0.024, 0.621±0.051, 0.961±0.005, 0.916±0.011 and 0.868±0.234 for the validation set, GSE30219 cohort, GSE50081 cohort and GSE8894 cohort, respectively, further supported its fine predictive performance. We then probed the survival value of the gene signature in the TCGA cohort and three other independent external cohorts, GSE8894 cohort, GSE50081 cohort and GSE30219 cohort (Figure 3A–3D). As demonstrated in Figure 3E, 3F, we demonstrated that high levels of risk scores were observed in relatively high TNM staging. However, there does not appear to be a necessary association between risk score and metastasis (Figure 3G).

### Investigation and validation of the hub genes associated with EMT

**Figure 4:**
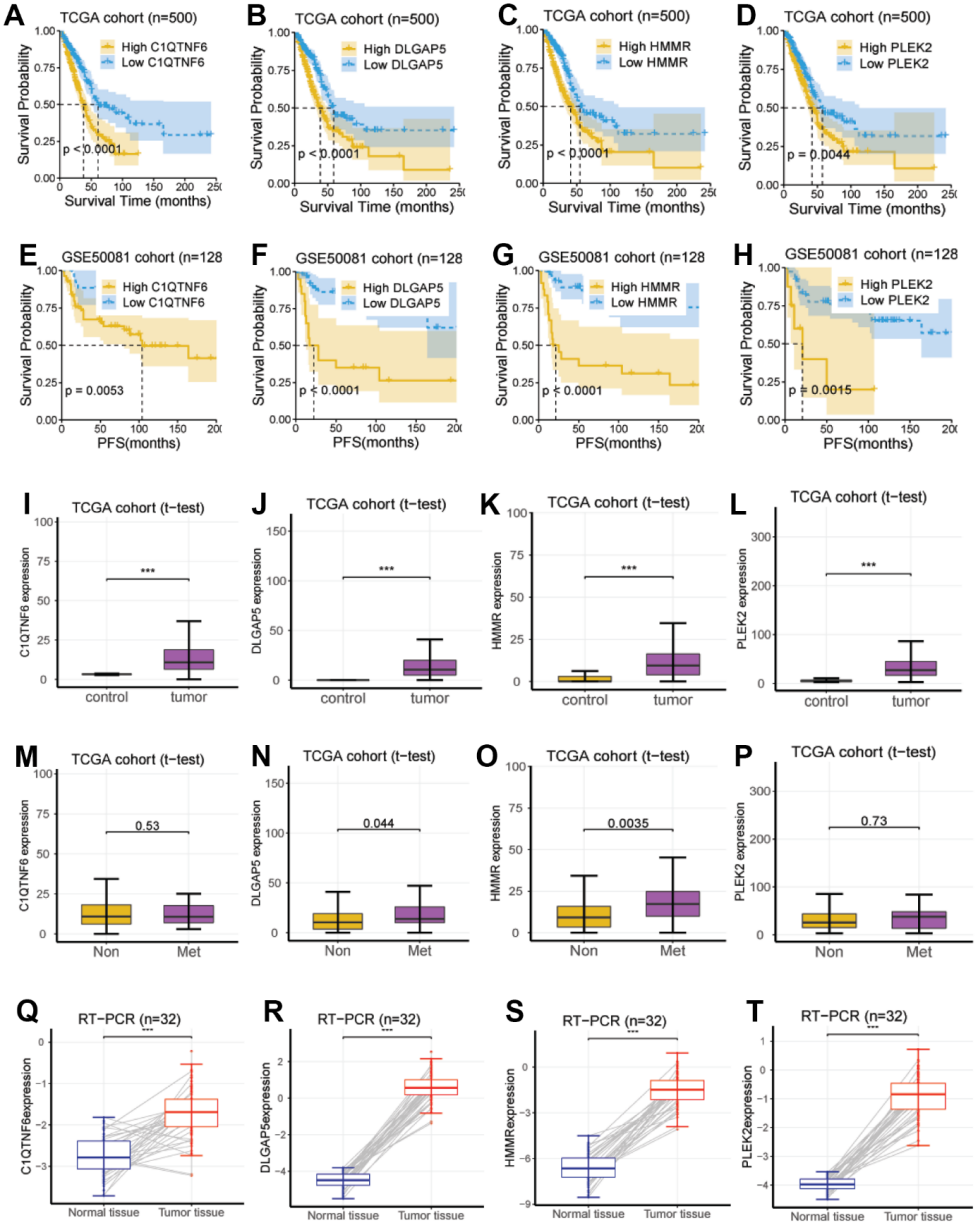
**Investigation and validation of the hub genes associated with EMT.** (**A**–**D**) Four genes, including *C1QTNF6*, *DLGAP5*, *HMMR*, and *PLEK2*, had a significant survival relevance for OS in the TCGA cohort (log-rank test; *P* < 0.01). (**E**–**H**) Four genes, including *C1QTNF6*, *DLGAP5*, *HMMR*, and *PLEK2*, had a significant survival relevance for PFS in the GSE50081 cohort (log-rank test; *P* < 0.01). (**I**–**L**) Consistent with the above findings, the expression of the four genes was enhanced in tumor tissues compared with normal tissues using RNA-seq from the TCGA cohort (*P* < 0.001). (**M**–**P**) *DLGAP5* and *HMMR* were significantly upregulated in the lung metastatic foci as compared with non-metastatic lung foci, while *C1QTNF6* and *PLEK2* were not. (**Q**–**T**) *C1QTNF6*, *DLGAP5*, *HMMR*, and *PLEK2* were also significantly upregulated in the cancerous tissues as compared with matched normal tissues using RT-PCR for 32 pairs of LUAD tissues and matched normal tissues from our hospital (*P* < 0.001).

We next investigated the eight genes (*CCNB1*, *PLEK2*, *DERL3*, *C1QTNF6*, *DLGAP5*, *HMMR*, *GJB3,* and *SPOCK1*), to identify the genes related to potential hub in LUAD development. We explored the survival significance of these genes in three independent cohorts (TCGA, GSE8894, and GSE50081). We demonstrated that four genes, including *C1QTNF6*, *DLGAP5*, *HMMR*, and *PLEK2*, had a consistent survival value in these three cohorts, while other genes have inconsistent performance in these cohorts, these four genes (*C1QTNF6*, *DLGAP5*, *HMMR*, and *PLEK2*) also had a survival relevance for PFS (log-rank test; *P* < 0.01; Figure 4E–4H).

Then, using RNA sequencing data from the TCGA cohort, we did so with the aim of determining their expression between lung metastases and non-metastatic lung lesions. According to the data we obtained, DLGAP5 and HMMR were significantly upregulated in pulmonary metastatic lesions, we also compared it with non-metastatic ones (P < 0.05; Figure 4N, 4O), whereas C1QTNF6 and PLEK2 were not (P > 0.05; Figure 4M, 4P).

At last, we performed RT-PCR using 32 pairs of LUAD tissues and 32 normal tissues matched from our hospital to further verify the reliability of the above results generated from RNA sequencing data.

### Establishment of a nomogram for precision medicine

**Figure 5:**
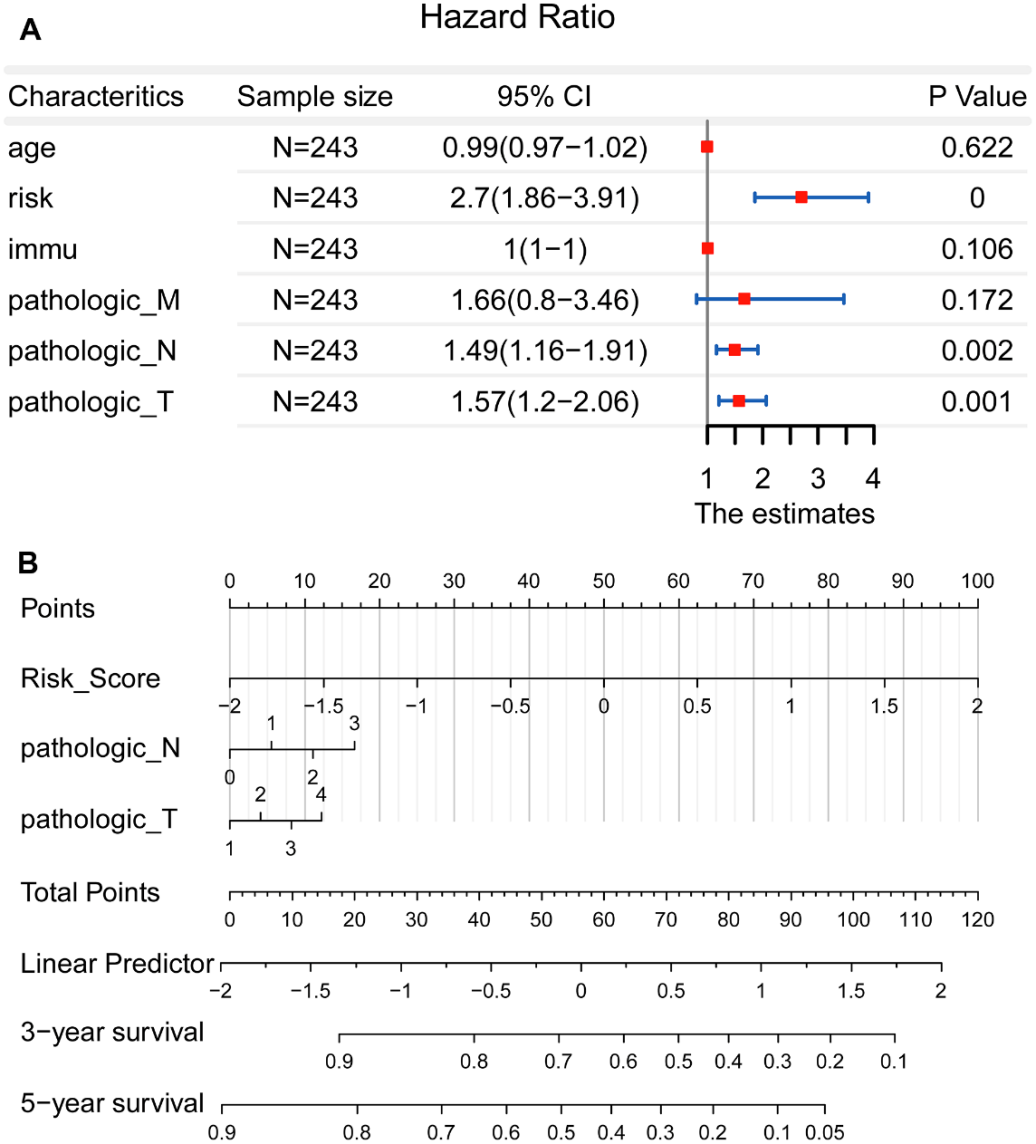
**Establishment of a nomogram for precision medicine.** (**A**) Forest plot showed that the risk score, pathological T, and pathological N could be used as effective prognostic characteristics for LUAD (*P* < 0.01). (**B**) A nomogram was constructed to accurately predict the patients’ survival risk for precision medicine.

However, there seemed no significant prognostic value in the factor of pathological M (*P* = 0.172). This may be opposite to the known knowledge, namely the patients of pathological M possessing poor survival. Therefore, the clinical data of 243 patients were further analyzed. It was shown that a total of 229 patients were in M0, meanwhile, there were 14 patients in M1. This suggested that the unbalanced sample size may affect the accuracy of the statistical results. At last, as shown in Figure 5B, a nomogram including pathological T, pathological N and risk score was constructed.

### Silencing of *PLEK2* reduces the proliferative and migrated properties of LUAD cells

**Figure 6:**
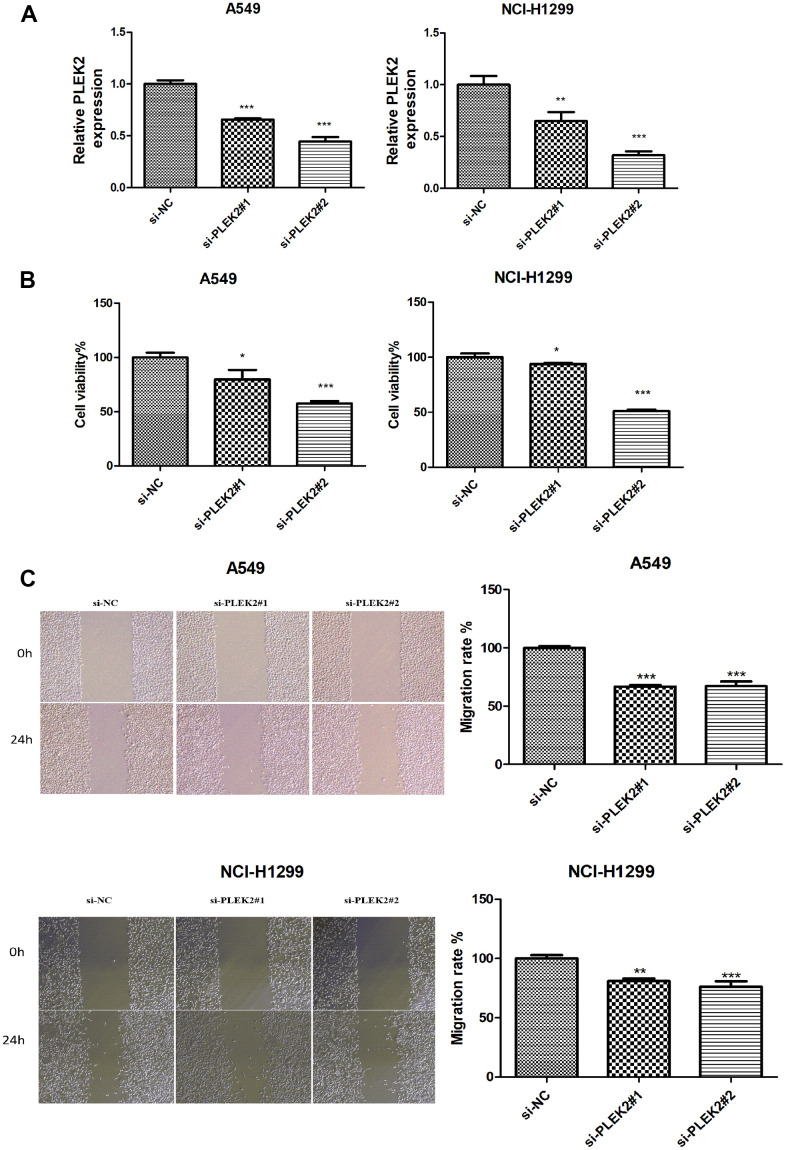
**Silencing of *PLEK2* reduces the proliferative and migration ability of LUAD cells.** (**A**) The expression of *PLEK2* after transfection of si-PLEK2#1, si-PLEK2#2 or si-NC in A549 and NCI-H1299 cells was detected via RT-PCR (*P* < 0.01), demonstrating that *PLEK2* was successfully knocked down in A549 and NCI-H1299 cells. (**B**) The viability of A549 and NCI-H1299 cells after transfection of si-PLEK2#1, si-PLEK2#2 or si-NC was assessed by CCK-8 assay (*P* < 0.05), demonstrating that decreased *PLEK2* could lead to a reduction in the proliferative ability in A549 and NCI-H1299 cells. (**C**) The migration of A549 and NCI-H1299 cells after transfection of si-PLEK2#1, si-PLEK2#2 or si-NC was calculated through wound healing assay (*P* < 0.01), demonstrating that decreased *PLEK2* could lead to a reduction in the migration ability in A549 and NCI-H1299 cells.

We took *PLEK2* as an example to explore its effect on LUAD progression *in vitro*. *PLEK2* was silenced in LUAD cells to determine the transfection efficiency firstly. As manifested in Figure 6A, si-PLEK2#1 or si-PLEK2#2 was transfected into A549 and NCI-H1299 cells, demonstrating that siRNA was successfully introduced into LUAD cells. CCK-8 assay showed that the viability of A549 and NCI-H1299 cells could be dramatically decreased by knocking-down *PLEK2* (*P* < 0.05; Figure 6B). Meanwhile, as illustrated in Figure 6C, the silenced *PLEK2* also prohibited the migrated capacities of A549 and NCI-H1299 cells (*P* < 0.01).

### Silencing of *PLEK2* prohibits the autophagy of LUAD cells

**Figure 7:**
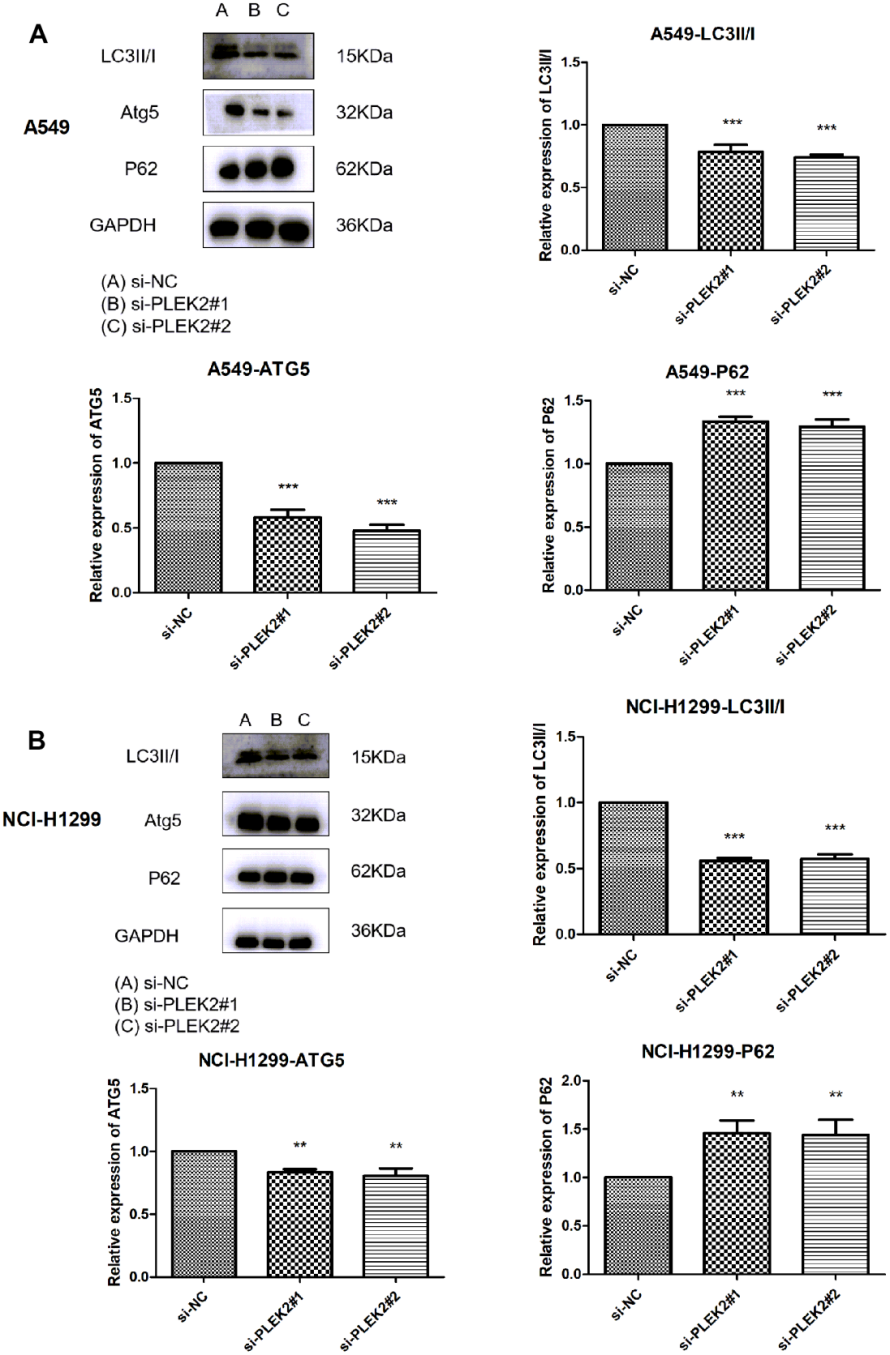
**Silencing of *PLEK2* prohibits the autophagy of LUAD cells.** (**A**) The protein levels of LC3II/I, ATG5 and P62 in A549 cells after transfection of si-PLEK2#1, si-PLEK2#2 or si-NC were determined by western blotting (*P* < 0.01), suggesting silencing of PLEK2 could critically inhibit the autophagy of LUAD cells. (**B**) The protein levels of LC3II/I, ATG5 and P62 in NCI-H1299 cells after transfection of si-PLEK2#1, si-PLEK2#2 or si-NC were determined by western blotting (*P* < 0.01), suggesting silencing of PLEK2 could critically inhibit the autophagy of LUAD cells.

When the cancer progresses to the advanced stage, autophagy can serve as a dynamic degradation role to lead to the growth of neoplastic cells through promoting metastasis. Therefore, the effects of *PLEK2* silencing on the autophagy of LUAD cells were then scrutinized. As shown in Figure 7A, knocking-down *PLEK2* remarkably reduced the protein levels of LC3II/I and ATG5, but elevated P62 protein amounts in A549 cells (*P* < 0.001). Similarly, in NCI-H1299 cells, silencing of *PLEK2* also suppressed the expression of LC3II/I and ATG5 and promoted P62 expression levels (*P* < 0.01).

### Autophagy activation reverses the suppressive impacts of *PLEK2* silencing on the migrated potentials of LUAD cells

**Figure 8:**
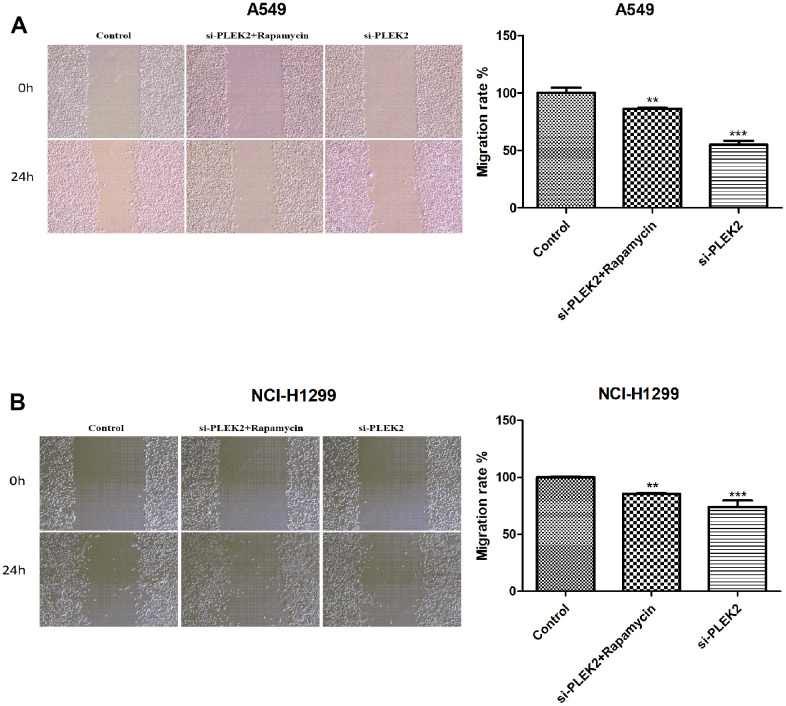
**Autophagy activation reverses the suppressive impacts of *PLEK2* silencing on the migrated potentials of LUAD cells.** (**A**) Wound healing assays showed that rapamycin treatment could reverse the suppressive impacts of *PLEK2* silencing on the migrated potentials of A549 cells (*P* < 0.01). (**B**) Wound healing assays showed that rapamycin treatment could reverse the suppressive impacts of *PLEK2* silencing on the migrated potentials of NCI-H1299 cells (*P* < 0.01).

To further probe the interplay of *PLEK2* with autophagy in the migratory progression of LUAD cells, A549 and NCI-H1299 cells that transfected with si-PLEK2 were incubated in the related environment. The time duration was 0.5 h. As indicated above, the silenced *PLEK2* exerted obviously inhibiting effects on cell migration in LUAD (*P* < 0.001; Figure 7A, 7B). However, the activation of autophagy significantly reversed the suppressive impacts of *PLEK2* silencing on the migrated potentials of A549 and NCI-H1299 cells (*P* < 0.01; Figure 8A, 8B).

## DISCUSSION

Lung cancer is one of the malignant tumors with high morbidity and mortality rates worldwide, posing a serious risk to patients’ quality of life and the health status of the population, and the 5-year survival rate after diagnosis of lung cancer is not high in most countries [13]. Lung adenocarcinoma is one of the most common histological types of lung cancer, and improvement of the prognosis of patients with lung adenocarcinoma is a keen concern for patients and society. In order to achieve this goal, there is an urgent need for more in-depth research and analysis of all aspects of lung adenocarcinoma. The complex tumor microenvironment in which the tumor grows and continues to progress plays a crucial role [14]. Therefore, it is important to explore specific biomarkers related to the tumor microenvironment and to study the therapeutic targets of the disease from the perspective of the tumor microenvironment for the treatment and prognosis of cancer patients.

In this study, we screened important tumor microenvironment-related genes in lung adenocarcinoma by bioinformatics and constructed an 8-gene tumor microenvironment-related prognostic risk model. Based on this prognostic risk model, we also explored the differences in immune infiltration density of immune cells in the tumor microenvironment and synthesized the potential prognostic features in the tumor microenvironment of lung adenocarcinoma, which will be helpful for prognostic assessment or development of new therapeutic targets for lung adenocarcinoma patients.

The first contribution of this report is the establishment of an EMT-related prognostic signature consisting of eight genes (CCNB1, PLEK2, DERL3, C1QTNF6, DLGAP5, HMMR, GJB3, and SPOCK1) to predict survival in LUAD patients. This EMT-related prognostic signature has three advantages. First, this genetic signature can predict not only OS but also PFS, which can be used as an effective tool for clinicians to accurately assess patients’ disease and give reasonable interventions.

Similarly, the study [15] pointed out that overexpression of C1QTNF6 was closely associated with poor prognosis in patients with LUAD. The study [16] reported that DLGAP5 was involved in related ovarian and non-small cell lung cancers. Consistent with our findings that this gene signature is associated with the cell cycle, silencing DLGAP5 leads to cell cycle arrest and inhibits the proliferation of NSCLC cells, and DLGAP5 shRNA inhibits cell migration and invasion *in vitro* [17]. In addition, overexpression of the hyaluronan receptor HMMR is associated with inflammation and poor prognosis in LUAD progression, and loss of HMMR can limit the ability to metastasize distantly [18, 19]. Mechanistically, the HCG18/miR-34a-5p/HMMR network is thought to trigger LUAD progression [20, 21]. It is well known that its prognostic value for PFS and OS has been rarely described. Therefore, we used PLEK2 as an example to further explore its effect on LUAD progression *in vitro*. As expected, we noted that the deletion of PLEK2 not only inhibited the proliferation and migration ability of LUAD cells, but also prohibited autophagy of LUAD cells. These results imply that PLEK2 may be a potential molecular target for LUAD therapy. Overall, these four key genes are upregulated in cancer tissues and have prognostic value for PFS and OS, and are key genes with potential.

Other studies have reached the same conclusion, showing that targeted therapy is significantly more effective than conventional regimens in the postoperative adjuvant treatment of patients with early-stage lung adenocarcinoma with EGFR gene mutations [22, 23]. As for targeted therapy, there is a lack of studies in patients with early-stage lung adenocarcinoma with the other gene mutations mentioned here. However, there is a challenge of secondary resistance to targeted therapies, especially the widespread emergence of secondary resistance to EGFR-TKI and ALK-tyrosine kinase inhibitors (ALK-TKI) by mechanisms that are mainly gene mutations, growth of resistant clones, or growth of alternative signaling pathways. The growth or activation of alternative signaling pathways, and genomic instability. Therefore, how to solve the problem of secondary resistance in targeted therapy is the focus of future research. Currently, there are no targeted drugs for TP53 mutations.

As scientists matured in their research on tumors, they gradually realized that the occurrence and development of malignant tumors are influenced by multiple factors. Therefore, it is important to study genes in greater depth and combine them with other small biomolecules. Therefore, we have been studying genes in greater depth and combining them with other biomolecules for a more accurate and precise assessment of patients with early lung adenocarcinoma. The study [24] established an EGFR impact score for the assessment and management of lung adenocarcinoma. In the assessment and management of lung adenocarcinoma, activation of the driver pathway should be assessed not only on the basis of individual hotspot mutations (e.g., EGFR L858R), but also on the basis of multiple mRNA expression profiles to predict the role of the EGFR pathway in lung cancer cells, and a study by EGFR-LEONG et al. showed the single-region whole-genome sequencing of 20 lung cancer cases. The study [25] concluded that 7-lncRNA 7-lncRNA signaling was also suggested to predict OS in patients with non-small cell lung cancer, especially those with early-stage tumors carrying wild-type KR. It has also been suggested that 7-lncRNA signaling can predict OS in patients with NSCLC, especially those with early-stage tumors carrying wild-type KRAS or EGFR.

A brief review was done through a comprehensive analysis of data from the GEO and TCGA cohorts. In addition, several key oncogenes and pathways of LUAD were identified. These findings provide a theoretical basis for the clinical treatment of LUAD.

There are some limitations in this study. First, the accuracy of the prognostic prediction model requires improvement. Usually, the AUC value above 0.8 is considered ideal. Second, more *in vivo* and *vitro* experiments are needed to deeply investigate the biological effects of *PLEK2* in lung cancer. Overall, future research should be directed towards these aspects.

## CONCLUSIONS

This study has successfully constructed and validated several epithelial-mesenchymal transition-related biomarkers in LUAD using bioinformatics and lab experiments. PLEK2 was used as an example to explore its effect on LUAD progression *in vitro* using RT-PCR, western blot, CCK8, si-RNA and wound healing assay. Silencing of PLEK2 was shown to reduce proliferative and migrated ability of lung cancer cells via prohibition of autophagy. These findings provide the rationale for further investigation and would aid clinical decision-making in LUAD immunotherapy.

## MATERIALS AND METHODS

### Human samples

In this experiment, we selected a total of 32 pairs of specimens, all of which were LUAD tissues, in addition to matched normal lung tissues from 32 patients with LUAD who underwent surgery in our hospital. Patients were diagnosed by histological examination and had not received any treatments before enrollment. The obtained samples were used for succeeding RNA sequencing. The experimental procedures performed in this investigation were authorized by our hospital’s ethics committee. Before admission, all individuals provided a written informed consent.

### Cell culture

They were cultivated with an added 10% FBS and then kept in an environment with 5% CO_2_ and a temperature of 37° C. After achieving 90% confluence, cells at passages three to five were utilized for transfection experiments.

### Cell transfection

PLEK2-siRNA#1/#2 (si-PLEK2#1/#2) and the corresponding negative control (si-NC) were acquired from Sangon Biotech (Shanghai, China). The transfection tests were conducted in strict compliance with the protocol. Briefly, si-PLEK2#1, si-PLEK2#2 and si-NC were introduced individually into related cells for 48 h with the aid of Lipofectamine 3000. Finally, the LUAD cells that had been successfully transfected were gathered for the subsequent experiments.

### CCK-8 assay

A549 and NCI-H1299 cells were inoculated into culture plates with 96-wells (4000 cells/well). Following incubation of 72 h, CCK-8 solution (10 μL) was pipetted into the wells before they were incubated for two more hours at 37° C. Cell viability was then determined using a microplate reader (Carl Zeiss, Oberkochen, Germany) with a 450nm filter.

### RNA isolation and quantitative real-time PCR

In strict accordance with TRIzol reagent’s protocols (Invitrogen, USA), total RNA from tumor tissues was isolated and then synthesized to cDNA productions via a GoScript Reverse Transcription System Kit (A5000; Promega, USA). PCR procedures were implemented with the aid of SYBR green Kit (Accyrate Biotechnology Co., Ltd., China) on an ABI 7500. The used primers were listed as follows: human β-Actin (F: CATGTACGTTGCTATCCAGGC, R: CTCCTTAATGTCACGCACGAT), *C1QTNF6* (F: CCACAGGACACAGGGTCAC; R: CTCAGAGTCACAGCACCGTT), *DLGAP5* (F: GGTTGTGAGGGTTCCTGCTT; R: TCAACGACGTGGGCATTACA), *HMMR* (F: TTCAGTTGTCGAGGAGTGCC; R: GGAGATGGTGCACAACCAGA), and *PLEK2* (F: AGGTGCGTCGCTTTGTTCTA; R: CAGACACGAGTGAACCACGA). β-Actin was used for normalization and the 2^-ΔΔCt^ approach was for calculating expression levels.

### Western blotting

Total proteins were extracted from A549 and NCI-H1299 cells (anti-LC3, anti-Atg-5, anti-p62 and anti-GAPDH; nanoTools, Munich, Germany; 1:1000 dilution) and then the secondary antibodies (Abcam, Cambridge, UK; 1:5000 dilution). Protein bands were visualized by Tanon5200 gel imaging system (Tanon, Shanghai, China) with corresponding ECL kit (Tanon), and quantified by Alpha Innotech software.

### Public data collection

We downloaded the data of RNA sequencing from UCSC Xena (https://xena.ucsc.edu/). This part of data includes the expression of 52124 genes for 585 lung tissues, including 526 cancerous tissues and 59 normal tissues, it is corresponding with survival data. Meanwhile, data for LUAD patients were also obtained from three independent GEO datasets (GSE8894, GSE50081, and GSE30219), which were used to validate the results generated from the TCGA cohort.

### Differentially expressed genes (DEGs) calculation

DEGs were calculated with the aid of R package “edgeR” as previously described. Meanwhile, DEGs (57 paired cancerous tissues and normal tissues) from TCGA cohort of the LUAD patients were estimated via a paired design, with the formula as follows: “design = model.matrix (~ patient + group)” in R.

Data that FDR < 0.001 and |logFC| > 2 were the selected criteria of DEGs.

### ssGSEA

We screened EMT gene set (526 tumor specimens) from LUAD patients’ TCGA cohort and with the aid of ssGSEA algorithm based on R package “GSVA” as previously described, the enrichment scores of these genes were calculated. Enrichment score is closely associated with the downregulation or upregulation of genes.

### EMT-associated gene signature model

A number of 500 samples (576 samples in total) possess the completed survival data, which is separated to 2 groups in random: validation set (n = 100) and training set (n = 400). Subsequently, variable (*P* < 0.05) was served as an input role in LASSO analysis. Finally, through multivariate Cox regression mode, we developed the prognostic EMT-associated gene signature with *P*-value of gene that less than 0.05 in LASSO analysis.

### EMT-associated gene signature validation

In the process of this research, we also use some related models as basis, what we use them for is to calculate the related scores of risks to quantify the prognostic signature. Meanwhile, Kaplan-Meier curve, C-index and AUC parameter in validation set, training set and three independent sets (GSE30219 cohort, GSE50081 cohort, and GSE8894 cohort) were used for the validation of predictive performance.

### Functional annotation

The implementation of functional annotation was carried out with the help of the relevant tool, the R package “cluster Profiler”, which is very useful as a full set of functional annotation tools to help relevant researchers in order to further their understanding of the biological significance associated with it.

### Nomogram development

In combination of LUAD patients’ clinical parameters with the obtained risk scores, a series of nomograms were built to assess the survival risk of LUAD patients more precisely and guide clinical practice at the same time.

### Statistical analysis

SPSS 25.0 statistical software was used. Differentially expressed genes were obtained by processing data with R language software 3.6.3 and limma software package. Survival curves were plotted using the Kaplan-Meier method, and the comparison of high and low expression groups of key genes was performed using Log rank *χ*^2^ test. Key genes with statistically significant differences in survival analysis were analyzed for differential expression using the ggplot2 and ggpubr software packages, and the Kruskal-Wallis test was used for multiple groups testing and the Wilcoxon test for two-pair testing. p < 0.05 was considered a statistically significant difference.

### Data availability

The experimental data used to support the findings of this study are available from the corresponding author upon request.

### Consent to publish

We agree with the publication of this paper.
